# Genomic Signatures of Selection Across Climate Gradients and a Geographic Mosaic of Coevolution in an Ant Social Parasite–Host System

**DOI:** 10.1093/molbev/msaf293

**Published:** 2025-11-11

**Authors:** Maide Nesibe Macit, Erwann Collin, Maria Esther Nieto-Blazquez, Marion Kever, Maria Litto, Esther Jaitner, Markus Pfenninger, Barbara Feldmeyer, Susanne Foitzik

**Affiliations:** Senckenberg Biodiversity and Climate Research Centre (SBiK-F), Molecular Ecology Group, Frankfurt am Main, Germany; Institute of Organismic and Molecular Evolution, Johannes Gutenberg University, Mainz, Germany; Institute of Organismic and Molecular Evolution, Johannes Gutenberg University, Mainz, Germany; Senckenberg Biodiversity and Climate Research Centre (SBiK-F), Molecular Ecology Group, Frankfurt am Main, Germany; Institute of Organismic and Molecular Evolution, Johannes Gutenberg University, Mainz, Germany; Institute of Organismic and Molecular Evolution, Johannes Gutenberg University, Mainz, Germany; Institute of Organismic and Molecular Evolution, Johannes Gutenberg University, Mainz, Germany; Senckenberg Biodiversity and Climate Research Centre (SBiK-F), Molecular Ecology Group, Frankfurt am Main, Germany; Senckenberg Biodiversity and Climate Research Centre (SBiK-F), Molecular Ecology Group, Frankfurt am Main, Germany; Institute of Organismic and Molecular Evolution, Johannes Gutenberg University, Mainz, Germany

**Keywords:** coevolution, social parasite, slave-making, *Temnothorax*, GWAS, transcriptomes

## Abstract

Coevolutionary dynamics in host–parasite systems are shaped by reciprocal selection and environmental context. When hosts and parasites share ancestry and ecological overlap, selection can act on similar traits, but population structure and geography may generate adaptive mosaics. Here, we present the first study to investigate genome-wide signatures of selection in response to a broad climatic gradient and a geographic mosaic of coevolution between a social parasite and its host. We examined these processes in the dulotic ant *Temnothorax americanus* and its congeneric host *Temnothorax longispinosus* using population genomics, genome-wide association analyses, and transcriptomics. Host populations showed very weak and parasites stronger population structure, enabling geographic mosaic dynamics. Genomic responses to parasite prevalence were divergent: Hosts showed signatures of selection on immune genes, whereas regulatory genes associated with raiding were under selection in parasites. Both species displayed convergent signatures of climate adaptation, including loci related to desiccation resistance, stress response, and parasite prevalence, with signals in communication and recognition genes involved in hydrocarbon biosynthesis, chemosensory perception, circadian rhythms, and venom production. Transcriptome analyses revealed contrasting patterns, with host gene expression linked to parasite prevalence and parasite expression more strongly shaped by climate. Together, our results reveal a genomic mosaic of coadaptation, in which population structure, asymmetric selection, and ecological variation interact to generate divergent yet interconnected evolutionary trajectories. Our findings highlight communication and recognition as recurrent arenas of antagonistic coevolution, underscore climate as a pervasive selective force, and establish a framework for investigating molecular coevolution in social parasite systems.

## Introduction

Antagonistic interactions between hosts and parasites can lead to dynamic coevolutionary arms races where both parties continuously adapt and diversify in response to one another (Dawkins and Krebs 1979; Hamilton 1982; Nash et al. 2008; Tellier et al. 2014). These arms races occur at the population rather than species level, with the independence of local interactions shaped by the extent of gene flow among populations. According to the geographic mosaic theory of coevolution (GMTC) (Thompson 1999, 2005), resulting arms races escalate at different rates across each mosaic piece, with variations in environmental conditions and community compositions leading to divergent local adaptations. Some traits may evolve in response to both antagonistic partners and abiotic conditions, where trade-offs may arise when both demands cannot be fulfilled simultaneously (Wolinska and King 2009; Amandine et al. 2022). This presents two interrelated challenges: (i) determining which observed changes in phenotypic traits result from the coevolutionary arms race versus environmental conditions and (ii) inferring the extent to which these processes are interdependent or linked through pleiotropic interactions (Thompson 1999, 2005). Such interactions can promote or constrain trait expression in antagonistic relationships, occasionally resulting in counterintuitive outcomes (Clarke and Fraser 2004; Gregory 2009; Wisz et al. 2013; Oppold et al. 2016; Mahmud et al. 2017). Species population structure, shaped by historical events and ongoing gene flow, influences evolutionary cohesion and the available adaptive genetic variation (Whitlock 2004; Durrett and Schweinsberg 2005; Greischar and Koskella 2007; Gupta and Vadde 2019). Consequently, it also shapes the adaptive evolutionary trajectories of these species in response to environmental and biotic selection pressures across their geographic ranges (Aaltonen 2002; Tigano and Friesen 2016). To determine the genomic basis of these interacting evolutionary forces, a comprehensive analysis must consider them simultaneously.

Brood and social parasitism is a lifestyle characterized by behavioral manipulation and exploitation of host social traits (Hughes et al. 2012; Jongepier et al. 2015; Jamie and Kilner 2017; Rojas-Ripari et al. 2021). Social parasites evade the costs associated with labor tasks such as brood care, nest construction, and foraging by exploiting their host's workforce (Lenoir et al. 2001; Buschinger 2009). Hosts can counterevolve a range of defenses to mitigate the fitness costs of parasitism, including avoidance behavior, aggressive defenses, and tolerance strategies limiting parasite-inflicted damages (Råberg et al. 2009; Feeney et al. 2014; Gibson and Amoroso 2022). Recognition is often a prerequisite for successful counteradaptation to detect and discriminate parasites from conspecifics or benign species (Langmore et al. 2011). For instance, hosts of avian brood parasites may evade parasitism by such identification and subsequent rejection of foreign, parasitic eggs (Soler 2017; Manna et al. 2019). Similarly, hosts of social insect parasites initiate defense responses upon identifying parasite-specific chemical cues (Delattre et al. 2012).

Such cue recognition is facilitated through odorant receptors located in the antennal sensilla of insects (Hölldobler and Wilson 1990; Ozaki et al. 2005). *Odorant receptor* genes, especially those of the 9-exon subfamily, have undergone expansion during the evolution of insect sociality, along with the emergence of complex chemical communication and the regulation of social organization (Engsontia et al. 2015; Slone et al. 2017; McKenzie and Kronauer 2018; Legan et al. 2021; Gautam et al. 2024). Social parasites have convergently lost many *odorant receptor* genes, particularly those linked to worker behavior (Caminer et al. 2023; Harrison et al. 2025) due to relaxed selection on traits less important in the parasitic lifestyle (Jongepier et al. 2022; Schrader et al. 2021; Gautam et al. 2024).

As chemoreceptors, these odorant receptors detect cuticular hydrocarbons (CHCs), a complex mixture of chemical compounds whose composition varies qualitatively and quantitatively (Sprenger and Menzel 2020). CHCs are critical for social insect communication, especially in nestmate recognition, enabling the discrimination of colony members from parasitic intruders (Dani et al. 2001; Blomquist and Bagnères 2010; Lorenzi et al. 2011). They also contribute to desiccation resistance by sealing the cuticle and supporting thermoregulation, playing an important role in climate adaptation (Menzel et al. 2018; Sprenger et al. 2019). CHC composition can change dynamically to alleviate drought stress (Sprenger et al. 2018) or to adapt to fluctuating temperatures (Wagner et al. 1998, 2001; Martin and Drijfhout 2009; Menzel et al. 2018; Sprenger et al. 2019), but may consequently impair detection efficiency (Wittke et al. 2022), hinting at a potential trade-off in fulfilling both functions. This highlights the challenge of optimizing enemy recognition and desiccation resistance, especially in species interactions across diverse climatic landscapes.

The myrmicine ant *Temnothorax americanus* is a dulotic social parasite, which raids *Temnothorax longispinosus* colonies to capture worker brood and exploits them for their social behaviors (Wesson 1939; Hölldobler and Wilson 1990; Buschinger 2009; Schmid-Hempel 2019; Rabeling 2021). Both species are widely distributed across northeastern North America, with parasites more abundant in southwestern regions (Jongepier et al. 2014; Macit et al. 2024). Geographic variation in parasite prevalence and thus selection pressures on local hosts prompted the evolution of divergent coadaptive traits. These include modifications in the CHC profile composition: *T. americanus* employs chemical insignificance by reducing its amount of recognition cues to evade host detection (Lenoir et al. 2001; Kleeberg et al. 2017; Kaur et al. 2019), while its host diversifies its colony-specific CHC profiles in areas where parasites are present, impairing advances at parasite chemically matching with hosts (Jongepier and Foitzik 2016; Kleeberg et al. 2017; Collin et al. 2025). Behavioral strategies in the host exhibit spatial shifts associated with variation in parasite abundance and climate (Jongepier et al. 2014; Segev et al. 2017; Collin et al. 2025): In heavily parasitized, warm regions, hosts tend to show reduced aggression toward highly aggressive parasites, whereas in colder regions with low parasite prevalence, hosts display greater aggression toward their less aggressive sympatric parasites (Segev et al. 2017; Collin et al. 2025). This pattern may reflect climatic constraints, with colder regions favoring stronger aggression due to shorter active seasons and limited resources, whereas warmer climates may promote alternative defensive strategies such as evasion (Sorvari and Hakkarainen 2004; Cristaldo et al. 2016; Segev et al. 2017; Collin et al. 2025), although these mechanisms require further investigation. Furthermore, hosts modify their brain transcriptome activity in response to encounters with aggressive parasites, while parasites show dynamic changes in their gene expression before and during attacks, corresponding to shifts in activity levels (Alleman et al. 2018; Kaur et al. 2019). Adding a spatial component, a previous Pool-seq study on the host *T. longispinosus* showed that patterns of antennal gene expression are strongly linked to local parasite prevalence (Macit et al. 2024). This study further provided the first insights on the genomic basis of population-level host adaptation, identifying strong selection on immune functions and olfactory perception, namely, selection on *peptidoglycan recognition protein* (*PGRP*) and various *odorant receptor* genes.

Fundamentally, the previously unrecognized link between parasite prevalence and local climate conditions that Macit et al. (2024) identified may generate and shape a geographic mosaic of coevolution across heterogeneous habitats. Variations in local parasite population size can create a selection mosaic on hosts, while climate acts as an overarching selective force on both species, critically influencing their behavior and chemical profiles (Segev et al. 2017; Collin et al. 2025). Because host and parasite are closely related and share nests, both experience similar environmental conditions, making climate adaptation a necessary baseline against which to detect signals of antagonistic coevolution. While convergence in traits associated with climate adaptation is expected, traits involved in host–parasite coadaptation may show partial or asymmetric overlap, with genetic redundancy enabling different genetic routes to the same phenotype (Barghi et al. 2019). For example, CHC genes linked to chain length may overlap due to roles in desiccation resistance (Sprenger et al. 2018) in both host and parasite, but divergence is expected in recognition traits, such as *n*-alkanes, where hosts increase profile diversity to reduce parasitism (Jongepier and Foitzik 2016; Kleeberg et al. 2017) and parasites may evolve chemical mimicry. Likewise, aggression may not involve shared loci, as hosts rely on biting and stinging their enemies for nest defense (Koenig and Moreau 2024a), whereas *T. americanus* shows less overt aggression and instead employs Dufour's gland secretions as a manipulative strategy to distract hosts (Brandt et al. 2006).

**Fig. 1.**
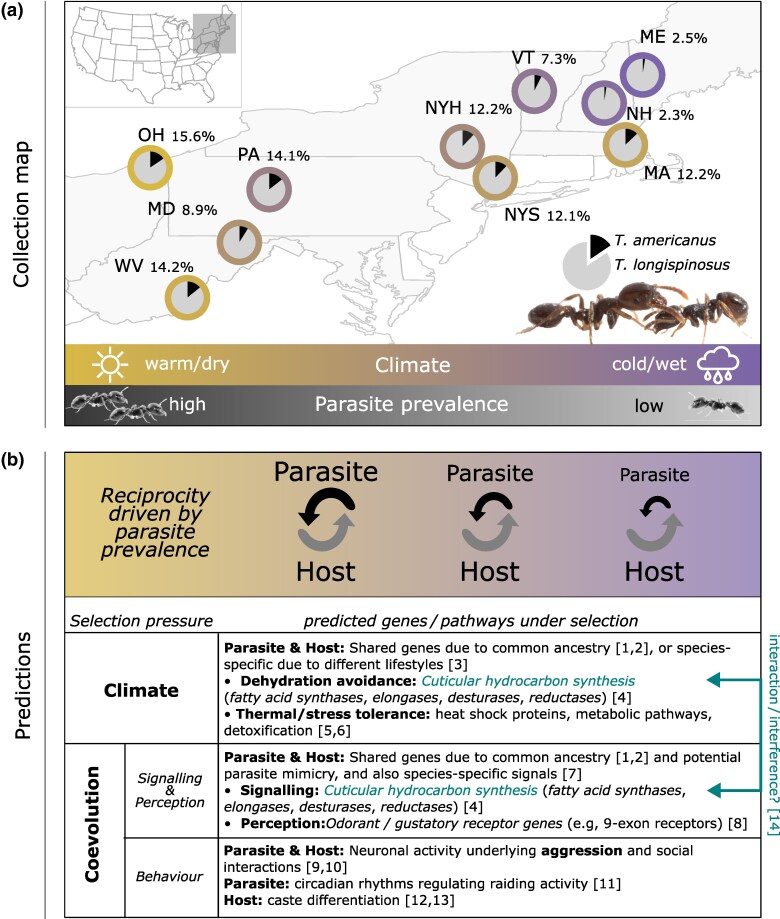
a) Map of collection sites across northeastern United States, named after US state abbreviations (exception: NYH, New York Huyck; NYS, New York South in the US state New York) based on Macit et al. (2024). Parasite prevalence (in %) is indicated next to each state abbreviation. Pie charts illustrate the proportion of parasitic *T. americanus* colonies relative to host *T. longispinosus* colonies. The surrounding ring represents local climatic conditions, summarized by PC1 eigenvalues, with yellow color indicating warm/dry and purple cold/wet climates (Macit et al. 2024; see Materials and Methods section). b) Conceptual overview of coevolutionary dynamics. Host reciprocity is depicted as fixed, whereas parasite reciprocity varies with local parasite prevalence. Climate is shown as an overarching factor influencing both species similarly. The accompanying table lists genes and pathways predicted to be under selection by climate and coevolution. Further, coevolution is broken down into key functions and phenotypes important in their coevolution. References: (1) Cini et al., (2015); (2) Feldmeyer et al., (2022); (3) Jackson et al., (2020); (4) Sprenger et al., (2019); (5) Farahani et al., (2020); (6) Liu et al., (2017); (7) Kleeberg et al., (2017); (8) Engsontia et al., (2015); (9) Alleman et al., (2018); (10) Kaur et al., (2019); (11) Feldmeyer et al., (2017); (12) Koenig and Moreau, (2024a); (13) Caminer et al., (2023); and (14) Wittke et al., (2022).

Here, we present the first genome-wide association study (GWAS) on the population-level genomic basis of coadaptation in a host and its social parasite, disentangling the effects of biotic and abiotic interactions across populations. This framework allows us to disentangle environmental from coevolutionary selection and highlights how similarities and differences between species may contribute to a geographic mosaic of coadaptation. We resequenced individual ants from over 120 colonies, each of the host *T. longispinosus* and its parasite *T. americanus*, across ten sites within their shared range in the northeastern United States (see Fig. 1a). These areas differ in climate and local parasite prevalence, likely creating a selection mosaic that is expected to shape patterns of reciprocity as well as drive selection on genes central to climate adaptation and host–parasite coevolution. For both species, we investigated: (i) population structure, (ii) genomic differentiation between populations, (iii) genome-wide associations with biotic and abiotic environmental variables (parasite prevalence and climate), (iv) genome-wide associations with key traits relevant to host–parasite interactions (chemical profiles, attack- and defense-related behaviors) obtained from our twin study (Collin et al. 2025), and (v) gene expression patterns in head and fat body associated with parasite prevalence and climate. We predict that genes critical in the coevolution of these species are involved in enemy recognition (e.g. odorant receptors), signaling (e.g. CHCs via fatty acid synthesis), behavior (e.g. biogenic amines), and as previously identified immune response (e.g. *PGRPs*; Macit et al. 2024) (see Fig. 1b for a conceptual overview of predictions). Genes underlying climate adaptation may be conserved either due to shared ancestry or parallel responses to common ecological factors. These may include heat shock proteins, metabolic and detoxification pathways, and, most notably, genes involved in CHC synthesis, which contribute to desiccation resistance (Fig. 1b; Sprenger et al. 2018). Our integrative approach establishes links between key phenotypic traits and their underlying genomic architecture, providing insights into the complex spatial dynamics of biotic and abiotic factors that shape coevolutionary patterns between social hosts and their parasites.

## Results

### Population Structure

**Fig. 2.**
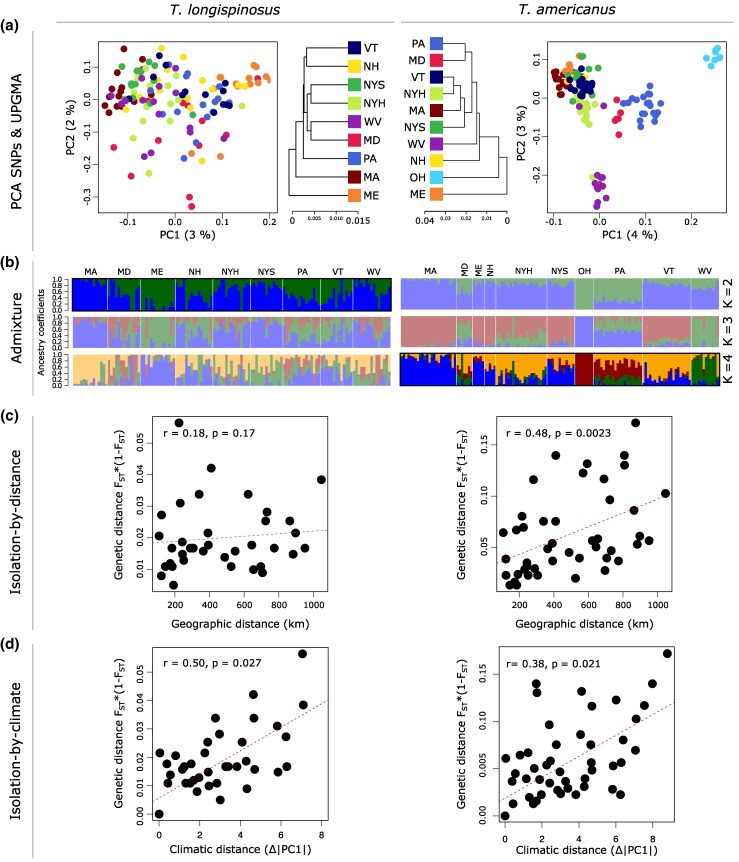
Population structure analysis. a) Principal component analysis of SNP data, based on a thinned dataset containing ∼50k SNPs per species. UPGMA tree based on pairwise genetic differentiation (F_ST_ values; see Figure S1) depicts clustering of populations according to their genetic similarity. Populations are encoded by colors. b) Admixture plot depicting the identified genotypes and their proportions for each population. Highlighted are admixture subplots for the lowest cross-entropy identified using *LEA()* ran 50× (Figure S2a). Colors represent the different genotypes identified per K. (c) Isolation-by-distance. (d) Isolation-by-climate.

We resequenced host and parasite individuals from ten populations across the northeastern United States (host, one individual from 15 independent colonies per population; parasite, one individual from 5 to 24 independent colonies per population) (Fig. 1a; S1). A principal component analysis of thinned single-nucleotide polymorphisms (SNPs) revealed no clear clustering in the host, but more distinct grouping in the parasite (Fig. 2a). Population structure in the host species was nearly absent, with very low pairwise F_ST_ values (mean F_ST_ = 0.021). In contrast, the parasite exhibited low but more than twice as high F_ST_ values (mean F_ST_ = 0.055; Figure S1). Accordingly, UPGMA (unweighted pair group method with arithmetic mean) trees revealed short branch lengths in the host species, consistent with low genetic differentiation between populations, whereas the more structured parasite species showed longer branches, reflecting stronger subdivision (Fig. 2a). Admixture analyses revealed two genotype clusters in the host, whereas four distinct clusters were detected in the parasite (Fig. 2b). Isolation-by-distance was stronger in the parasite (Mantel test: *r* = 0.48; *P* = 0.0027) than in the host (*r* = 0.18; *P* = 0.17; Fig. 2c). By contrast, isolation-by-climate, measured as the absolute difference in climate PC1 eigenvalues (see Materials and Methods section), was found to be strong in the host (*r* = 0.50; p = 0.027) and the parasite (*r* = 0.38; p = 0.021; Fig. 2d). Additional details are provided in the SI (Additional Results and Discussion—Population Structure).

### Genomic Variations Linked to Population Differentiation

**Fig. 3.**
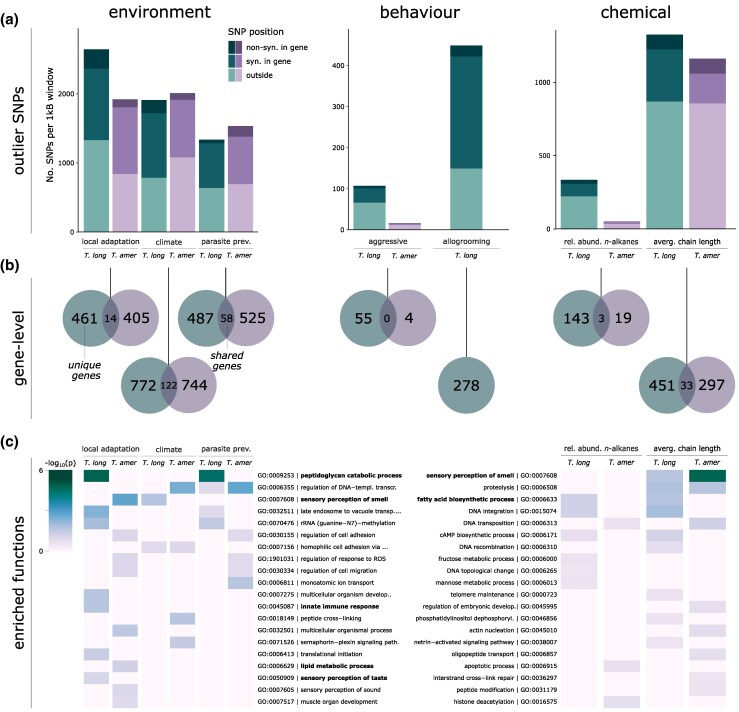
Genome-wide association analyses. a) Total number of unlinked SNPs (i.e. one SNP per 1 kb windows) identified by the different analyses (“local adaptation” refers to loci differentiated between populations), divided into ns-SNPs, syn. in-exon SNPs and SNPs outside of gene regions (see Figure S4a for more information). b) Number of unique genes in which these SNPs reside, with overlap indicating shared (i.e. orthologous) SNP-containing genes in both species (see Figure S5b for more information). c) Heatmap of a subset of enriched biological GO terms (p < 0.05; −log_10_(p) > 1.3 as depicted in the legend by the color gradient) with candidate functions in bold.

We used *OutFLANK* (Whitlock and Lotterhos 2015) to identify differentiated loci (p ≤ 0.05) based on F_ST_ outliers, indicative of local differentiations between populations. We detected 6,018 outlier loci in the host, including 282 nonsynonymous SNPs (ns-SNPs) across 461 genes (Fig. 3a and b; Table S3). In contrast, the parasite exhibited only half as many outlier SNPs (3,250), but the number of ns-SNPs (177) and associated genes (405) did not differ significantly from those of its host (p_adjust_ > 0.05 for both; Figures S4a and S5a). Candidate genes in the host included *insulin-degrading enzyme* (*IDE*), *chitinase 10* (*CH10*), and several *PGRP* genes (Table S4). In the parasite, notable genes included *cubilin* and *vitellogenin 1-like* and genes involved in CHC synthesis, such as *fatty acyl-CoA reductase* and *desaturases* (Table S5). Among the 12 enriched biological functions of locally adapted host genes (Fisher's exact test, p ≤ 0.05) were immune-related processes, particularly the “peptidoglycan catabolic process.” In the parasite, 20 enriched biological functions of locally adapted parasite genes included pathways related to lipid metabolism, such as the “lipid metabolic process” (Fig. 3c; S2).

### Genomic Variations Linked to Environmental Traits

We used *BayPass* v2.2 (Gautier 2015) with its covariate model to associate genotypes with environmental factors (parasite prevalence and climate) and phenotypic traits (chemical profile and behavior) in the host and the parasite (summary in Table S3, raw data in S2).

#### Parasite Prevalence

**Fig. 4.**
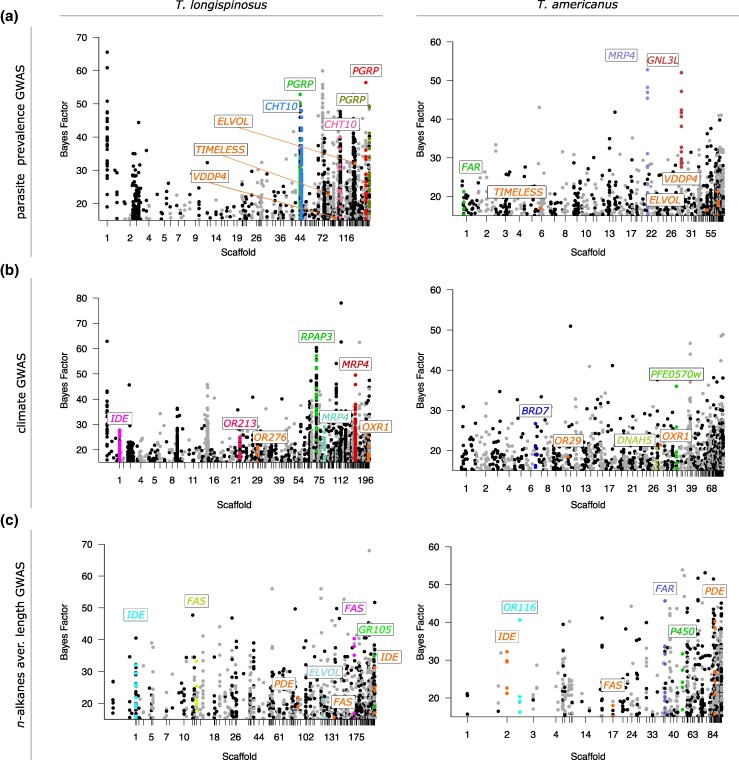
GWASs of *T. longispinosus* and its parasite *T. americanus*. Manhattan plots display significant SNPs associated with a) parasite prevalence, b) climate, and c) average chain length of *n*-alkanes. Only SNPs exceeding a BF of 15 and located within or proximal (±2 kb) to annotated genes are shown. Shared (i.e. orthologous) candidate genes between the two species are highlighted in orange (Table S6). Candidate genes presented here represent a subset of those exhibiting a high number of ns-SNPs with high BF values. More details can be found in Tables S4 and S5.

Parasite prevalence, defined as the relative abundance of parasites in relation to host colonies, is a proxy for parasite success and the selective pressure parasites impose on local host populations. In the host, we identified 2,706 significant SNP loci (with Bayes factor (BF) ≥ 15) associated with population-wide parasite prevalence (including 155 ns-SNPs) across 487 genes. In contrast, we identified fewer SNPs in the parasite (1,690 SNPs, including 54 ns-SNPs), but those were distributed across more genes (525; gene count: χ^2^ = 10.66, p_adjust_ = 0.0019; Fig. 3a and b; Figure S5a). Host candidate genes were similar to those identified to be differentiated between populations, including *PGRP* genes (highest BF > 50) and multiple *CHT10* genes (highest BF = 39; Fig. 4a; Table S4). These genes were also identified in a previous host Pool-seq GWAS analysis (Macit et al. 2024 ; Figure S6), further supporting their role in parasite resistance/tolerance. We found that heterozygosity of significant loci within *PGRPs* was mainly differentiated between populations ANOVA (Analysis of variance) *F* = 2.65; *P* < 0.007; Figure S3). In contrast, parasite genes associated with parasite prevalence showed little overlap with those identified to be differentiated among populations. Among the ones with the strongest signals were two *guanine nucleotide-binding protein-like 3* genes (*GNL3L*; highest BF > 50), two *multidrug resistance-associated protein 4* (*MRP4*; highest BF > 40), and a *fatty acyl-CoA reductase* (*FAR*; highest BF = 21; Fig. 4a; Table S5). Enriched functions differed between host and parasite: In the host, again, immune-related pathways such as the “peptidoglycan catabolic process” were among the five most enriched functions, while among the enriched functions in the parasite were those relating to transcriptional regulation, such as the “regulation of DNA-templated transcription” (Fig. 3c; S2).

#### Climate

By using population-specific climate data encompassing various temperature and precipitation variables (see Macit et al. 2024), we identified 3,016 SNPs associated with climate in the host (including 192 ns-SNPs) across 772 genes (Fig. 3a and b). In the parasite, we identified a similar number of associated SNPs (2,582) and genes (774) but fewer ns-SNPs (97; ns-SNP count: χ^2^ = 9.94, p_adjust_ < 0.003; gene count: p_adjust_ > 0.05; Figures S4a and S5a). Host genes with the strongest genomic associations included two *multidrug resistance proteins* (*MRP4*; highest BF > 40), a *trichohyalin-like* gene (highest BF > 50), and an *odorant receptor* gene (*OR*; highest BF = 23.9; Fig. 4b; Table S4). In contrast, parasite candidate genes showed significantly weaker associations than their host (BF: ANOVA *F* = 20.41, p_adjust_ < 0.001; Figure S4b). Among the parasite genes with the strongest associations were *MATH* and *LRR domain-containing protein* (*PFE0570w*; highest BF = 36.0), *x-ray repair cross-complementing protein 5* (highest BF = 27.5), and a *fatty acid reductase* (*FAR*; highest BF = 21.3; Fig. 3b; Table S5). Host climate-associated genes were enriched for five biological functions, primarily related to gene regulation and posttranslational modifications (“regulation of DNA-templated transcription,” “protein phosphorylation”). In contrast, the parasite's 14 enriched functions were distinct from its host's, with several related to metabolic and catabolic processes and signaling pathways (e.g. “semaphorin-plexin signaling pathway”; Fig. 3c).

#### Convergent Patterns of Adaptation

We used *OrthoFinder* v.2.5.4 (Emms and Kelly 2015) to infer orthology among genes of both species, allowing us to identify shared genes that were differentiated among populations and climate adapted or correspond to parasite prevalence. We identified 14 shared genes differentiated between populations in the host and the parasite, including *pumilio homolog 2*, linked to spatial memory in *Mus musculus* (Siemen et al. 2011), and *retinol-binding protein pinta-like*, related to visual responses in *Drosophila* (Wang and Montell 2005; Table S6). However, the number of shared genes was not higher than expected by chance, indicating population differentiation acting on different genes and pathways in both species (hypergeometric model *P* = 0.22; Jaccard index = 0.021; Figure S5b). In contrast, for both parasite prevalence and climate, the number of shared candidate genes between the two species identified in the GWAS was greater than expected by chance, indicating selection on the same genes (both *P* < 0.0001; Jaccard indices: climate = 0.10; parasite prevalence = 0.068). In the GWAS on parasite prevalence, 58 shared genes were found under reciprocal selection in both species. Many of these genes were associated with neural, synaptic, and developmental functions (*neurotrimin*, *semaphorin-1A* and *semaphorin-2A*, *fasciclin-1, latrophilin/cirl, netrin-A*), lipid modifications and homeostasis (*elongation of very long chain fatty acid protein*, *phosphodiesterase*), circadian rhythm (*timeless*), and venom production (*venom dipeptidyl peptidase 4*; Fig. 4a; Table S6). In the climate GWAS, we identified 122 shared genes associated with climate adaptation in both species, twice as many as in the parasite prevalence GWAS. Several of these genes were associated with functions related to neural and photo- and chemosensory perception (*fascicilin-1*, *semaphorin-2A*, *phospholipase A1, OR*), but also to oxidative and environmental stress management (*oxidation resistance protein 1*, *carboxylic ester hydrolase*), and water retention (*nephrin*; Fig. 4b;Table S6).

### Genomic Variations Linked to Behavior

Interactions between dulotic parasites and their hosts are mediated via behavioral traits, particularly aggression during raids. As hosts typically recognize parasites by their chemical profile, encounters often escalate to overt aggression due to chemical mismatches, suggesting targeted aggressive responses are subject to selection. Here, we use the number of aggressions after introducing an individual parasite into the host nest as a behavioral proxy (Collin et al. 2025). We detected only a weak genomic signal associated with aggressive behavior in both species. For the host, we identified 131 associated SNPs (including six ns-SNPs) across 55 genes, which included *protein groucho* (BF = 22.7), *calpain-D* (BF = 16.5), and also *insulin-degrading enzyme* with several intron SNPs (highest BF = 15.8; Table S4). Those genes were enriched in four biological functions, such as neural repair and perception (“response to axon injury,” “visual perception”). For the parasite, we identified only 15 SNPs (including two ns-SNPs; Table S5) within four genes, significantly less than host candidate genes (gene count: χ2 = 36.42, p_adjust_ < 0.001; Figure S5a), which included a *juvenile hormone esterase* (BF = 15.6). Due to the low number of genes, no enriched functions could be determined (Table S5, S2).

Host workers are often injured during raids, with their nestmates typically reacting by wound grooming. To simulate parasite-induced injuries, we observed nestmate responses to leg removal in host colonies, hypothesizing grooming behavior to be linked to parasite prevalence (see First Aid in Ants in SI). Instead, we found a stronger association with local climate, with higher allogrooming frequencies in colonies from warmer regions (χ^2^ = 7.36; *P* = 0.007; Figure S14), which we explained as preventing bacterial proliferation in warmer temperatures. We identified strong genomic associations with this behavior, with 523 associated SNPs (including 27 ns-SNPs) across 278 genes (S2). Candidate genes included *neprilysin-4* (BF = 24.3) and *DNA topoisomerase 3-alpha-like* (BF = 19.5; Table S4). Among the 12 enriched biological functions were some involved in transcription and expression (“regulation of DNA-templated transcription,” “regulation of gene expression”; Table S4).

### Genomic Variations Linked to the CHC Profile

The composition of CHCs plays an important role in host–parasite interactions, with methyl-branched alkanes serving as recognition signals. However, chemical traits are also involved in climate adaptations, particularly through the protective role of long-chain linear *n*-alkanes against dehydration (Sprenger et al. 2019). As CHC biosynthesis is mediated via a conserved pathway, similar genes may be subject to selection in both species. However, we found no associated genetic variants in either species with recognition cues identified by Collin et al. (2025). Contrarily, for the relative abundance of linear *n*-alkanes, we identified 424 associated SNPs in the host (including 27 ns-SNPs) across 143 genes, and considerably fewer in the parasite, with only 56 SNPs (including three ns-SNPs) across 19 genes (Fig. 3a and b). Candidate genes in the host included some known to be involved in CHC biosynthesis, such as *fatty acid synthases* (*FAS*; highest BF > 40) and *cytochrome P450* genes (hereafter referred to as *P450*; highest BF = 39), along with olfactory perception genes such as two *ORs* (highest BF = 22), which included one belonging to the 9-exon subfamily, and a *gustatory receptor* gene (*GR*; BF = 19; Table S4). The parasite exhibited fewer associated genes (χ2 = 79.03; p_adjust_ < 0.001; Figure S5a), including a *FAS* (BF = 25.5) and a *P450* gene (BF = 15.9), but no perception genes (Table S5). Among the eight enriched functions of host candidate genes were processes related to CHC biosynthesis, and meta- and catabolic processes (“fatty acid biosynthetic process,” “fructose/mannose metabolic process”). The parasite had half as many enriched functions, including those relating to gene regulation and cell death (“histone deacetylation,” “apoptotic process”), but none relating to CHC biosynthesis. Two shared genes were found between the species, of which one was annotated as *cullin-3* (Table S6).

The average chain length of linear *n*-alkanes can further influence desiccation resistance properties. We found that the average chain lengths of linear *n*-alkanes differed among populations in both species (host, ANOVA *F* = 3.50; parasite, *F* = 3.67; both *P* < 0.01) but also between species, with the host having longer linear *n*-alkanes (Mann–Whitney U test, *z* = 13.62; *P* < 0.00001; Figure S10; see Additional Results and Discussion—Chemical Characteristics in SI). Using chain lengths as a parameter, we identified 2,216 SNPs (including 100 ns-SNPs) across 481 host genes in our GWAS. The parasite had a similar number of associated SNPs (2,114) as well as ns-SNPs (102) with similar association values (ns-SNP count and BF: both p_adjust_ > 0.5; Figure S4a and b). However, the parasite had fewer candidate genes than the host (297; gene count: χ^2^ = 15.19, p_adjust_ < 0.0005; Figure S5a). Identified host candidate genes were those known in CHC biosynthesis, such as several *FAS* genes (highest BF > 40), a *very long chain fatty acid protein* (BF = 21.1), and again perception genes such as an *OR* (BF = 15.5) and *GR* (BF = 18.9; Fig. 4c; Table S4). The parasite showed similar genes, such as several *P450* (highest BF = 39.5), *FAS* (highest BF = 18.0), and *FAR* genes (highest BF > 40), as well as several *ORs*, two of which belonged to the 9-exon subfamily (highest BF = 33.1; Fig. 4c; Table S5). We identified 15 shared candidate genes between the two species, more than expected by chance (Jaccard coefficient = 0.063; *P* < 0.001; Figure S5b), which included a *FAS* and a *phosphodiesterase* gene (Table S6).

### Transcriptional Activity Associated With Parasite Prevalence

**Fig. 5.**
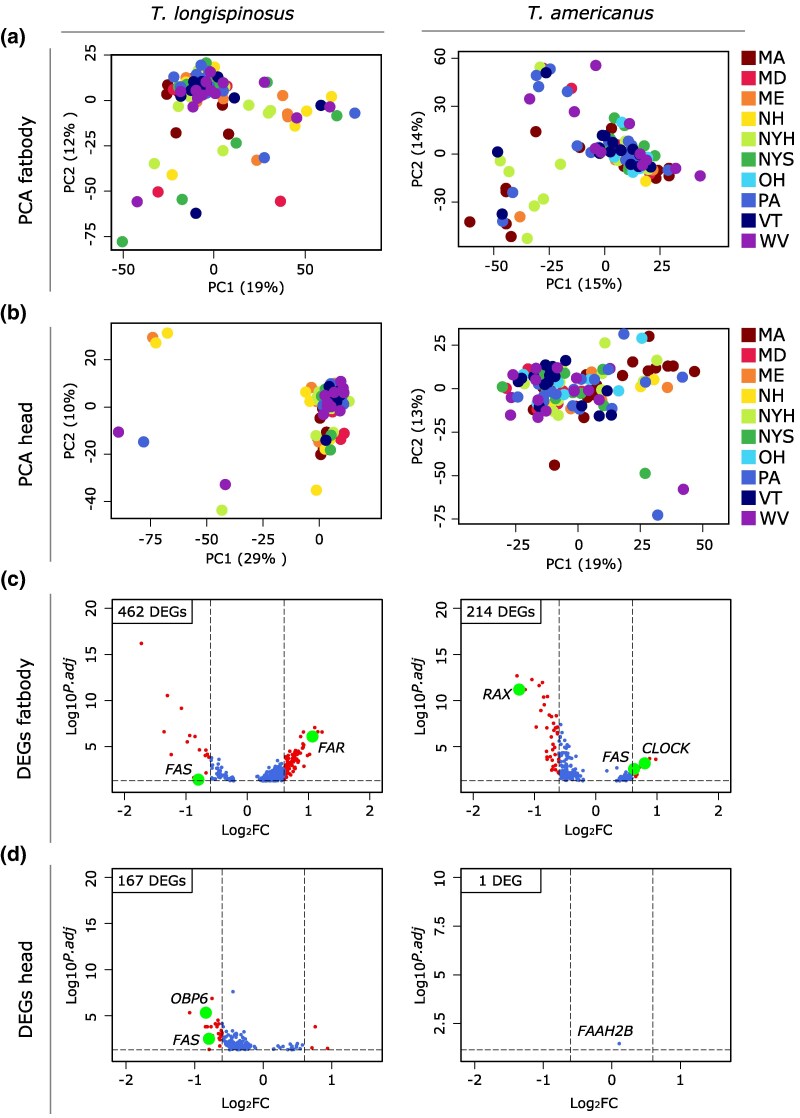
Analysis of transcriptome data. a) PCA of fat body transcriptome and b) head transcriptome in both species. c) Volcano plot of differentially expressed genes associated with parasite prevalence in the fat body and d) in the head samples, with curated candidate genes with high or low log_2_FC highlighted.

Our earlier study on host population pools revealed a strong correlation between global antennal gene expression and local parasite prevalence (Macit et al. 2024). Compared to that earlier study, which was conducted after only a few weeks of standardized ant husbandry, we kept ants used for this study for over 8 months, so that workers might have emerged under standard laboratory conditions. We also used individual-level data from both species to test whether transcriptional activity in the head (which includes the chemosensory antennae and the brain involved in processing and behavior) and in the fat body (a key physiologically active organ also involved in CHC synthesis) was similarly associated with local parasite prevalence (S1). Principal component analysis revealed no clustering according to populations (Fig. 5a and b), and none of the principal components, as a proxy for global gene expression, were associated with parasite prevalence in either species (Pearson's correlation in both tissues: *P* > 0.05). Using parasite prevalence as a continuous variable in a *DESeq2* analysis, we identified a greater number of differentially expressed genes in the host compared to the parasite in both tissues (fat body, χ2 = 92.19; head, χ2 = 162.91; both p_adjust_ < 0.001). The expression of 462 genes in the host fat body was associated with parasite prevalence, including a *FAR* gene and a *FAS* gene (Fig. 5c). In contrast, only 214 genes in the parasite fat body transcriptome were associated with parasite prevalence, approximately half the number observed in the host. Significant genes included a *FAS*, a *circadian clock-controlled gene* (*CLOCK*), and a *retinal homeobox protein Rx2* (*RAX*; Fig. 5c). Among the 14 enriched functions of candidate genes in the host fat body were those related to meta- and catabolic processes (S1). Fat body-expressed genes in the parasites showed 22 enriched functions, primarily associated with neuronal and stress management. In the host head transcriptome, the expression of 167 genes was associated with parasite prevalence, including a *FAS* gene and an *odorant-binding protein* (*OBP*; Fig. 5d). Among the 13 enriched functions were primarily those in fatty acid and metabolic processes (S1). Only one significant gene was found for the parasite's head transcriptome (*fatty acid amide hydrolase*; Fig. 5d).

Using the same transcriptome data, we analyzed associations between constitutive gene expression and climate. In the fat body, more genes were climate associated in the parasite (884) than in the host (525). In comparison, the head transcriptome revealed fewer linked genes, with similar numbers between the host (49) and parasite (40). Details, including gene functions, are provided in the supplement (Figure S11; Additional Results and Discussion in SI and S1).

### Parasite-Associated Loci Linked to Differential Gene Expression

Variants within noncoding regions can be important for adaptation if they lead to altered expression of the associated genes (Nourmohammad et al. 2017). We employed an Expression quantitative trait loci-like strategy to link SNPs identified in the parasite prevalence GWAS within regulatory regions (i.e. introns or ±2 kb upstream, referred to as 2 kb SNPs) to regulatory changes associated with parasite prevalence. We identified two genes, *multidrug resistance protein* (*MRP4*) and *CHT10*, which are differentially expressed in the host fat body and head transcriptomes, respectively. *MRP4* contained 90 intron SNPs, and *CHT10* contained 68 intron SNPs and two 2 kb SNPs associated with parasite prevalence. In the parasite head transcriptome, the expression of *fatty acid amide hydrolase* (*FAAH*) associated with parasite prevalence was found to contain ten intron SNPs and one 2 kb SNP (S1).

## Discussion

Coevolutionary divergence of key phenotypes in host–parasite interactions results from reciprocal selection pressures (Tellier et al. 2014; Kurtz et al. 2016 ), paired with selection pressures imposed by their shared environments. In species coinhabiting widespread heterogeneous environments, disentangling the genomic basis of the resulting mosaic of coevolution requires an integrative approach that accounts for the ecological context in which coevolution unfolds. Here, we examined the coevolutionary basis of the dulotic ant *T. americanus* and its host *T. longispinosus* across a broad climatic gradient in northeastern North America. By combining GWASs of environmental and phenotypic traits, and investigating gene expression, we explore both the genetic targets and regulatory architecture underlying host–parasite coadaptation.

### Disparate Population Structures Shape the Evolutionary Trajectories of Host and Parasite

Population genetic analyses revealed a marked contrast between weakly genetically structured host and more strongly genetically structured parasite populations. While this result was mainly based on genome-wide F_ST_ values, which are relative measures, with cross-species comparisons potentially being problematic (Meirmans and Hedrick 2011), both species were sampled from the same set of populations, analyzed with the same bioinformatic pipeline, and share ancestry, which makes relative differences in their genetic structure at the very least informative. Moreover, our results align with previous studies based on neutral genetic markers (Brandt et al. 2007; Pennings et al. 2011; Pamminger et al. 2014; Macit et al. 2024). This asymmetry could result in population-specific host–parasite trait combinations, where strong variation in parasite prevalence (2% to 16%) and climatic conditions (ranging from warm/dry to cold/wet) suggests that coevolutionary dynamics are unlikely to be uniform but instead vary regionally, consistent with predictions of the GMTC (Thompson 2005). Although we observe weak population structure in the host *T. longispinosus*, this does not contradict the geographic mosaic framework, which requires spatially variable selection, not necessarily strong genome-wide population structure. The detection of climate- and parasite-associated SNPs in the host therefore supports locus-specific adaptation despite low genome-wide differentiation. Thus, while the parasite exhibits a pronounced mosaic pattern across populations, the host participates in a more fine-scaled or transient mosaic, reflected in allele-frequency shifts at selected loci rather than broad demographic structure.

Phenotypic regional variation in behavior and chemistry identified in earlier studies in both species (Foitzik et al. 2001; Brandt and Foitzik 2004; Foitzik et al. 2009; Jongepier et al. 2014; Kleeberg et al. 2015, 2017) corroborate the GMTC, even though there is evidence that parts of the variation in behavioral and chemical traits may be explained by climate (Collin et al. 2025). Environmental heterogeneity across the study area may therefore generate an intertwined selection mosaic that influences where reciprocal adaptations are more or less likely to occur. Further, the parasite's likely recent range expansions into previously unparasitized regions (Jongepier et al. 2014; Macit et al. 2024), combined with low dispersal and small effective population sizes, may have enhanced drift and local adaptation (Whitlock 2004 ; Excoffier et al. 2009 ). Such demographic constraints may also underlie instances of parasite maladaptation in colder regions (Collin et al. 2025), reducing parasite adaptive potential. Consequently, low parasite prevalence in these areas may not impose sufficient selection pressure to elicit reciprocal host responses. This could result in coevolutionary cold spots in accordance with GMTC predictions, which could be formally tested for by directly comparing the strength of selection among populations.

### Genome-Wide Variation in the Host Is Associated With Parasite Prevalence

Genome-wide association analyses revealed differences between the two species in their genomic responses to parasite prevalence. In the host, we identified many associated loci indicating a highly polygenic basis, while in the parasite, fewer loci were involved. Candidate genes in *T. longispinosus* showed enrichment in immune and structural defense functions, notably *PGRPs*, which typically initiate antimicrobial responses (Dziarski 2004; Harris et al. 2015; Wang et al. 2019). Some *PGRPs* were also found to modulate behavior via neuroimmune interactions using the gut–brain axis (Gonzalez-Santana and Diaz-Heijtz 2020; Fioriti et al. 2024) and modulate egg-laying behavior via octopaminergic neuronal circuits (Kurz et al. 2017), suggesting pleiotropic links between immunity and socially relevant traits. Such dual functionality may reflect genomic trade-offs or pleiotropic interactions between pathogen defense and behavioral plasticity, warranting further investigation on their precise mechanisms in social insects. Ongoing research suggests an expansion in *PGRP* genes in the genus *Temnothorax*, followed by a secondary reduction in their social parasites (pers. com. Lumi Viljakainen). Moreover, a recent comparative genomics study showed that *T. longispinosus* possesses around twice the number of *PGRPs* than *T. americanus* (Vizueta et al. 2025). Additional strong signs of selection on *CH10*, involved in cuticle fortification, might indicate parasite-driven mechanical defenses against injuries inflicted during raids (Shahabuddin and Kaslow 1993; Qu et al. 2021; Rabadiya and Behr 2024).

In contrast, in the parasite, fewer loci were statistically linked to parasite prevalence. This may be because parasite prevalence reflects not only local success but is also strongly influenced by population structure, population size, demographics, various fitness parameters, and, importantly, local climatic conditions. These factors can lead to weaker statistical correlations (Vellend et al. 2014) and more diffuse associations (Abdellaoui and Verweij 2021). However, it may also reflect a rather homogeneous host population structure, which in turn may lead to a more homogeneous selection pressure of the host on the parasite, resulting in a weaker overall signature. Enriched functions of the candidate genes were related to gene regulation, suggesting that these adaptations are linked to dynamic changes in transcriptional activity. Strong signals of selection were still observed in a few candidate genes, for instance, in *MRP4*, which was previously found to be downregulated during parasite raids (Alleman et al. 2018). As a key regulator of prostaglandin signaling, this gene was previously linked to reproductive and behavioral regulation in solitary and social insects (Stanley 2006; Stanley and Kim 2019; McAfee et al. 2024). As such, it may have a role in the behavioral shift from an inactive (queen-like, egg-laying) to an active (forager-like, raiding) lifestyle during raiding season (Blatrix and Herbers 2004; Pohl and Foitzik 2011). Another candidate gene with strong selection signals was *guanine nucleotide-binding protein-like 3*, a G-protein associated with learning, memory, and pheromone production (Guillén et al. 1990; Calkins et al. 2019; Liu et al. 2021), which may facilitate scouting and coordination during raids.

Shared signatures of selection in both species were found in genes related to mechanosensation (*latrophilin/cirl*; Scholz et al. 2015), circadian rhythms (*timeless*), and venom production. These genes may play critical roles in mediating host–parasite encounters by detecting colonies or intruders, coordinating raids and defenses, and modulating aggression in response to each other. Selection on time-tracking genes may align with seasonal shifts in raiding and defensive behaviors (Brandt et al. 2005; Pamminger et al. 2011), contributing to the temporal organization of antagonistic phenotypes. The function of venom-related genes under selection in both species may likely diverge: Hosts employ venom in nest defenses (Koenig and Moreau 2024b), while parasites use glandular secretions from Dufour's gland in offensive behaviors such as host manipulation (Brandt et al. 2005; Jongepier et al. 2015). These genes, which are under selection in both species but may serve different functions, support the “toolkit hypothesis,” which proposes that coevolving partners repurpose conserved pathways to meet distinct ecological requirements (Cini et al. 2015; as predicted in Fig. 1b). While our analyses are purely correlational, further experiments are needed to validate the role of these candidate genes, as indeed (i) being fitness relevant in the according context and (ii) playing a role in host–parasite interactions. Experimental approaches such as CRISPR/Cas, now established for ants (Konu et al. 2023), and RNAi combined with behavioral assays offer powerful tools to directly test candidate gene functions and link SNP variants to causal effects on key traits.

### Climate Adaptation Shows Parallel Genomic Responses

As ectotherms, insects depend on physiological and biochemical adaptations to cope with climatic variation. Both species exhibit substantial overlap in climate-associated selection signatures, with shared genes and pathways potentially mediating convergent physiological responses. These included genes with functions in water retention (*nephrin*; Solanki et al. 2021) and stress responses (*oxidation resistance protein*; Wei et al. 2021), but also neuronal development (*semaphorin-2A*; Bates and Whitington 2007), which may pleiotropically modulate desiccation tolerance and climate-sensitive behaviors (Segev et al. 2017). The intertwined relationship between climate and parasite prevalence is evident in climate-induced behavioral shifts during host–parasite encounters (Collin et al. 2025), which can enhance, constrain, or redirect behavioral adaptations central to their coevolution under varying climatic conditions. Similar patterns have been reported in other systems, where abiotic conditions interact with biotic pressures to shape parasite dynamics through behavioral, ecological, or evolutionary pathways (Zamora-Vilchis et al. 2012; Møller et al. 2013; Bennett et al. 2016; Cable et al. 2017; Dziuba et al. 2023; Gray and Rabeling 2023). Social insects may be particularly sensitive to local climatic conditions, which can profoundly influence their host–parasite dynamics. In our focal system, both behavior and chemical phenotypes, as key traits in coevolution, are strongly shaped by climate in hosts and parasites alike (Segev et al. 2017; Collin et al. 2025). This highlights climate as a potential major driver of coevolutionary dynamics in social insects, a pattern likely extending to other social parasites. For example, climate change has shifted the migratory arrival times of avian hosts and their social parasites at shared breeding sites, causing phenological mismatches that may reduce parasite success (Saino et al. 2009; Mikula et al. 2024).

Species-specific climate-adaptation patterns, however, also highlight their distinct life history demands: Host-specific genes relate to cuticle maintenance and growth (*trichohyalin-like*, *IDE*; Stoppelli et al. 1988; Galagovsky et al. 2014), supporting long-term climatic resilience preferable for foraging, while parasite-specific genes are linked to development, metabolism, and CHC biosynthesis (*elongases*, *reductases*; Day et al. 2005; Sieron et al. 2019; Chougule et al. 2020; Farina et al. 2022), pointing to structural and physiological adjustments for their sedentary lifestyle.

### Behavioral Traits Show Weak Genomic Associations

Aggressive behavior plays a central role in both host defense and parasite offense during raids, yet it exhibits few detectable genomic associations in either species. This likely reflects the complex and indirect genetic architecture of behavioral traits, which can obscure clear links in genotype–phenotype association studies (Abdellaoui and Verweij 2021). Alternatively, gene expression plasticity may play a greater role than genomic divergence, as evident in substantial expression changes during raids in both species (Alleman et al. 2018). The *IDE* gene was upregulated in hosts during raids (Alleman et al. 2018) and was also among the few aggression-related host candidate genes. Since older foragers are the primary defenders during parasite attacks (Koenig and Moreau 2024a), and *IDE* has been implicated in age-dependent role shifts in *T. longispinosus* (Caminer et al. 2023), this gene likely links aggression to the transition to defending foragers in hosts.

In contrast, among the few parasite-specific aggression genes was *juvenile hormone esterase*, linked to pheromone degradation and odor perception (Wei et al. 2021), likely enhancing sensitivity in detecting host aggression cues, but also caste-shifts (Mackert et al. 2008), similarly linking age-dependent shifts to aggression. In contrast, host-specific allogrooming of injured workers showed strong genomic associations. This may be explained by the multifunctional role of allogrooming, including hygiene and social immunity, resulting in strong selection pressures that result in equally strong genomic signatures, similar to those identified in *Drosophila* (Yanagawa et al. 2020). Selection on chemosensory genes (*odorant* and *gustatory receptor* genes) associated with allogrooming underscores the importance of recognizing chemical signals from (injured) nestmates (Sambandan et al. 2006; Yanagawa et al. 2010, 2014) and nestmate recognition or discrimination more generally (Donaldson et al. 2025). Enrichment of regulatory functions further suggests dynamic mediation of this behavior, as also observed in honeybees (Hamiduzzaman et al. 2017).

### Genetic Basis of Chemical Signaling and Its Link to Chemosensory Perception

The CHC profile in social insects serves dual functions in desiccation resistance and chemical communication (Sprenger et al. 2019) and is likely shaped by climate and host–parasite coevolution. The abundance of (linear) *n*-alkanes and their average chain lengths, both traits linked to desiccation resistance, showed strong genomic associations in many genes in both species, hinting these to be complex polygenic traits. Similar genes and functions were selected for in both species, including *fatty acid synthases*, implicated in the CHC biosynthesis, highlighting the use of convergent genetic pathways. The selection of chemosensory genes such as *ORs*, especially those of the 9-exon subfamily serving pivotal functions in insect communication (Slone et al. 2017; Legan et al. 2021; McKenzie and Kronauer 2018; Gautam et al. 2024), and *GRs* in both species highlights the intricate pleiotropic relationship between chemical signaling and odorant perception. While previous *Hymenopteran* CHC genetic studies did not identify SNPs within perception genes (Buellesbach et al. 2022; Cohen et al. 2023; but Hartke et al. 2021), analyses on desiccation tolerance in *Drosophila* similarly found variants within *odorant receptor* and *gustatory receptor* genes (Griffin et al. 2017; Rajpurohit et al. 2018). This tight evolutionary coupling of perception and signaling in both species may support a putative signaling function of linear *n*-alkanes, as previously identified in social wasps (Michelutti et al. 2018), or alternatively reflect indirect effects on methyl-branched *n*-alkanes (i.e. key recognition cues) through their influence on CHC viscosity (Gibbs 1995; Gibbs and Pomonis 1995; Kleeberg et al. 2017). More broadly, a finely tuned communication system is essential in social insects, ensuring colony cohesion while simultaneously serving as a central target for parasite manipulation. Comparable dynamics occur in avian brood parasitism, where mimicry of parasitic eggs challenges host visual recognition, driving vision-based counteradaptations (Stoddard and Hauber 2017). Such parallels underscore that antagonistic coevolution in social host–parasite systems often centers on manipulation and detection of signals within shared channels of communication, with the genomic coupling of CHCs and odorant receptors in ants representing one clear manifestation of this general principle.

Using population-level data, we previously identified genomic loci associated with putative recognition cues in the host (Macit et al. 2024). However, we could not reproduce these results using colony-level data for both species, potentially due to high variability in individual recognition cues and limited statistical power from low sample sizes. popGWAS approaches (Pfenninger 2025) could offer improved power for detecting genomic associations in such variable traits and should be considered in future studies.

### Transcriptional Activity and Gene Regulation in Host–Parasite Interactions

Host defense and parasite raiding behaviors are accompanied by complex transcriptional changes (Alleman et al. 2018; Kaur et al. 2019). To assess population-level variation in constitutive gene expression, we analyzed transcriptomes from both species’ fat body and head tissues after 8 months of standardized laboratory conditions. While this design reduces environmental noise and allows for controlled comparisons, it captures baseline, adaptive expression and may overlook important context-dependent regulatory responses as were identified in previous studies (Alleman et al. 2018; Kaur et al. 2019). Thus, the presented data reflect evolved, genetically encoded expression patterns rather than acute, plastic responses to the antagonists. The most important ecological factor explaining differences in constitutive gene expression at the population level varied between species. In the host *T. longispinosus*, more differentially expressed genes were associated with local parasite prevalence, whereas in the parasite *T. americanus*, more were associated with climate. In the host, this pattern likely reflects a regulatory adaptation to selection imposed by raids from its parasite. Transcriptomic analyses of pooled host antennae samples showed gene expression strongly correlated with parasite prevalence but not climate (Macit et al. 2024). This is especially relevant since constitutive gene expression is costly and usually evolutionarily constrained (Wagner 2007). This may hint at the tremendous selective pressure of parasite prevalence on this tissue, where directional selection of genotypes responsible for this beneficial gene expression pattern leads to rapid fixation within highly parasitized populations (Ghalambor et al. 2015; Campbell-Staton et al. 2017; Rivera et al. 2021).

Expressed genes in the host associated with parasite prevalence included *odorant-binding protein* and *fatty acid synthase*, involved in chemical perception and CHC biosynthesis, linking transcriptional variation directly to recognition and signaling, both relevant in host–parasite interactions. In the parasite, the lower number of expressed genes associated with parasite prevalence may reflect both its sedentary lifestyle and simpler CHC profile (Kleeberg et al. 2017; Collin et al. 2025), but also the fact that local prevalence likely reflects a more diffuse selective force on the parasite than on its host. Among these few differentially expressed genes associated with parasite prevalence were *FAAH*, involved in lipid signaling and neural plasticity (Ueda et al. 2000; McKinney and Cravatt 2005; Sang and Chen 2006), a circadian regulator linked to time-sensitive behavioral cycles, and *retinal homeobox protein*, implicated in olfactory learning (Belyaeva et al. 2009; Hofmann et al. 2016). These genes suggest gene regulation in the parasite to be more dynamic, context dependent, and likely activated during raids. This highlights the role of timed gene expression, as evident in the importance of time-tracking genes in social parasite evolution (Feldmeyer et al. 2017), and points to flexible rather than constitutive regulatory strategies in the parasite.

## Conclusions

This study provides novel insights into how an ant social parasite and its host, despite shared ancestry and ecological overlap, may follow divergent genomic trajectories of coadaptation across multiple populations spanning a broad interaction range. Contrasting population structures, weak in the host and pronounced genetic structuring in the parasite, create a geographic mosaic in which coevolutionary dynamics and reciprocity can vary across space. An important conclusion from our study is that, although we identified species-specific genomic associations, the two interacting species responded in similar ways to environmental selection pressures, sharing a greater number of genes associated with climate, parasite prevalence, and a key signaling trait, CHC chain length, than expected by chance. Species-specific associations with parasite prevalence revealed links in the host to antibacterial immune genes, despite the parasite being social rather than microbial. Also, traits such as chemical profile composition and behavior showed few to no shared loci between species, consistent with our hypothesis that these traits follow more species-specific adaptive trajectories, as expected for polygenic traits. Constraints arising from the parasite's limited evolutionary potential in low-density populations may further shift the balance of the arms race in favor of the host. Strong links between chemical signals and *odorant receptor* genes highlight communication and recognition as recurrent arenas of antagonistic coevolution, while climate emerges as a pervasive selective force acting on both species. By integrating genomic, phenotypic, and environmental data, this study suggests potential for a geographic mosaic of coadaptation shaped by population structure, reciprocal selection, and ecological context. The host's greater evolutionary potential suggests stronger responses to climate change, while shared ancestry predicts that both species will continue to rely on overlapping genes in adaptation to environmental factors. Unexpected findings, such as the host's use of immune genes against social parasite pressure and the genomic interplay between signaling and perception genes, underscore the complexity of these interactions and the potential for further discoveries.

## Material and Methods

### Sample Collection and Estimation of Parasite Prevalence

Colonies of *T. longispinosus* and its social parasite, *T. americanus*, were collected from ten locations across the northeastern United States (Fig. 1a; Tables S1 and S2). Sampling occurred near roads and tracks in state parks and on private property, with permissions obtained. Ants were brought to Mainz, Germany, and maintained under standard laboratory conditions for 8 months before dissection. The accompanying behavioral and chemical data from Collin et al. (2025) were obtained 4 additional months later. Parasite prevalence, calculated as the percentage of parasite colonies within the local *Temnothorax* community, was determined using long-term collection data (Herbers and Foitzik 2002; Brandt and Foitzik 2004; Achenbach and Foitzik 2009; Jongepier et al. 2014; Kaur et al. 2019; Macit et al. 2024).

### Sample Preparation, Sequencing, and Pre-Processing

On average, 15 independent colonies per population and species were sampled (host, min = 14 max/mean/median = 15; parasite, min = 5, max = 24, mean = 15, median = 18). DNA from the thorax and RNA from the fat body and head from individual ants were extracted. Whole-genome sequencing (WGS) and RNA sequencing (RNA-seq) were performed on an Illumina NovaSeq 6000 platform by Novogene with a sequencing depth of ∼20×. Both were quality checked with *FastQC* v.0.11.9 (Andrews et al. 2015) and trimmed using *Trimmomatic* v.0.39 (Bolger et al. 2014). WGS data were mapped using *BWA mem* v.0.7.17 (Li et al. 2009), and RNA-seq data were mapped using *HISAT2* v.2.1.0 (Kim et al. 2015) to high-quality reference genomes for both species (NCBI IDs: *T. longispinosus*: GCA_048541765.1, *T. americanus*: GCA_048541705.1; Vizueta et al. 2025). For WGS data, variants were called using *BCFtools* v1.16 (Li, 2011) using custom filtering parameters (see Materials and Methods—Whole-Genome Analysis in SI), resulting in similar numbers of SNPs in both species (host, 1,677,757 SNPs; parasite, 1,604,099 SNPs). Transcript read count tables were generated using *HTSeq* v.2.0.2 (Putri et al. 2022) from RNA-seq data and used for *DESeq2* v.1.42.0 (Love et al. 2014) in *R* v.4.3.2 (R Core Team 2021) using parasite prevalence as a continuous variable. The analysis was repeated using climate PC1 values (obtained from Macit et al. 2024) as a continuous variable (Additional Results and Discussion—Differentially Expressed Genes Associated with Climate and Shared Gene Expression in SI). We defined candidate genes as those containing SNPs associated with the respective environmental variable and phenotypic traits identified in the GWAS (BF > 15), or those showing differential gene expressions associated with environmental parameters in the RNA-seq data (FDR; False discovery rate; p < 0.05). Further information on those candidate genes was collected using *InterPro* v.5.61.93 for functional annotation (Paysan-Lafosse et al. 2023), retrieving Gene Ontologies (GOs) to perform an enrichment analysis with *topGO* v.2.54.0 (Alexa and Rahnenfuhrer 2017) and *BlastP* v.2.13.0 (Altschul et al. 1990), searching against the nonredundant invertebrate database (retrieved on NBCI in January 2022), and proteomes of *Drosophila melanogaster* and *Apis mellifera* (retrieved on UniProt in January 2024; proteome IDs: UP000000803 and UP000005203; UniProt Consortium 2018). Any gene names given in this study refer to the best blast hit (based on e-value) generated in *A. mellifera* if not stated otherwise. Orthologs were identified using *OrthoFinder* v.2.5.4 (Emms and Kelly 2015). Chi-square tests in *R* assessed significant differences in candidate gene numbers (gene numbers: *T. longispinosus*, 16,064; *T. americanus*, 14,128) and ns-SNPs (exon lengths: *T. longispinosus*, 21,924,724; *T. americanus*, 20,133,265). BFs of ns-SNPs were log transformed and analyzed for differences between species using a one-way ANOVA in *R*. Significant overlaps in orthologous (i.e. shared) candidate genes were determined using the *hypergamous()* function from *SciPy* (Virtanen et al. 2020). All statistical tests were corrected for multiple testing using a significance threshold of Benjamini–Hochberg FDR-corrected p ≤ 0.05.

### Population Structure, Local Adaptation, and GWAS

Population structure was analyzed using the *R* package *sambaR* (De Jong et al. 2021), incorporating the *findstructure()* function. The lowest cross-entropy was observed at k = 2 for the host and k = 4 for the parasite (Figure S2a). Locally differentiated SNPs (p ≤ 0.05) were identified using *OutFLANK* (Whitlock and Lotterhos 2015) within the *selectionanalyses()* function in *sambaR*. GWASs were conducted using *BayPass* v2.2 (Gautier 2015) in its standard covariate mode. The parameters employed to identify their genomic basis included (i) parasite prevalence; (ii) climate parameters; (iii) chemical profiles (relative abundance of recognition cues, (linear) *n*-alkanes, and their average chain length); (iv) aggressive behavior, important for both parasitic raids and host defenses; and (v) allogrooming for the host. Other statistically significant species-specific behaviors, such as host brood-carrying and parasite passive behavior, were similarly analyzed but presented in the SI only. Data for chemical and behavioral analyses largely originated from Collin et al. (2025) from sister ants genotyped in this study. Identified SNPs were considered significant at a BF of ≥15 (Stefan et al. 2019). A detailed description of the methods used in this study can be found in the SI.

## Supplementary Material

msaf293_Supplementary_Data

## Data Availability

The following Supplementary material is available under open access: Supplements S1, S2, and S3 and Supporting Information (including Supplementary Tables, Figures, Methods, Results, and additional Discussion). All code supporting this study has been archived on Zenodo and is publicly available at doi.org/10.5281/zenodo.17048183. Raw sequence data were uploaded to the European Nucleotide Archive (ENA) and are accessible under study accession no. PRJEB76961.
